# Supplementary material for: Ultrasonographic Evaluation of Skin Toxicity Following Radiotherapy of Breast Cancer: A Systematic Review
Source: Int J Environ Res Public Health. 2022 Oct 18;19(20):13439. doi: 10.3390/ijerph192013439 (PMC9603505; doi:10.3390/ijerph192013439)
Supplement: Supplementary file 1 [file ijerph-19-13439-s001.zip › ijerph-1875750-supplementary.pdf]

Ultrasonographic evaluation of skin toxicity following radiotherapy of breast cancer: A systematic review

Table S1. Search terms used in the search strategy.

**Database**	**Search string**	**Results**
**Stage 1**		
**PubMed**	(((((breast cancer) OR (breast neoplasm)) OR (breast lesion)) AND ((((radiotherapy) OR (radiation therapy)) OR (radiation treatment)) OR (irradiation))) AND ((((((ultrasound) OR (ultrasonic)) OR (ultrasonography)) OR (ultrasonographic)) OR (sonography)) OR (sonographic))) AND (((((skin toxicity) OR (skin reactions)) OR (skin side effects)) OR (skin changes)) OR (dermatitis))	**259**
**Scopus**	(TITLE-ABS-KEY(breast AND cancer) OR TITLE-ABS-KEY(breast AND neoplasm) OR TITLE-ABS-KEY(breast AND lesion)) AND(TITLE-ABS-KEY (radiotherapy) OR TITLE-ABS-KEY (radiation AND therapy) OR TITLE-ABS-KEY (radiation AND treatment) OR TITLE-ABS-KEY (irradiation)) AND(TITLE-ABS-KEY (ultrasound) OR TITLE-ABS-KEY (ultrasonic) OR TITLE-ABS-KEY (ultrasonography) OR TITLE-ABS-KEY (ultrasonographic) OR TITLE-ABS-KEY (sonography) OR TITLE-ABS-KEY (sonographic)) AND(TITLE-ABS-KEY (skin AND toxicity) OR TITLE-ABS-KEY (skin AND reactions) OR TITLE-ABS-KEY (skin AND side AND effects) OR TITLE-ABS-KEY (skin AND changes) OR TITLE-ABS-KEY (dermatitis))	**127**
**Stage 2**		
	Reference lists of the included studies in stage 1	**386**
	included	**1**

Table S2. Quality check.

Quality Assessment from the National Heart, Lung, and Blood Institute for Observational Cohort and Cross-Sectional Studies																	
Criteria	(Borm et al., 2021)	(Landoni et al., 2013)	(Garnier et al., 2017)	(Wang et al., 2020)	(Torres et al., 2016)	(Lin et al., 2019)	(Yoshida et al., 2012)	(Yoshida et al., 2011)	(Liu et al., 2010)	(Liu et al., 2008)	(Keskikuru et al., 2004)	(Wratten et al., 2002)	(Wratten et al., 2000)	(Warszawski et al., 1998)	(Schack et al., 2016)	(Wong et al., 2011)	(Wratten et al., 2007)
1. Was the research question or objective in this paper clearly stated?	Y	Y	Y	Y	Y	Y	Y	Y	Y	Y	Y	Y	Y	Y	Y	Y	Y
2. Was the study population clearly specified and defined?	Y	Y	Y	Y	Y	Y	Y	Y	Y	Y	Y	Y	Y	Y	Y	Y	Y
3. Was the participation rate of eligible persons at least 50%?	Y	Y	Y	Y	Y	Y	Y	Y	Y	Y	Y	Y	Y	Y	Y	Y	Y
4. Were all the subjects selected or recruited from the same or similar populations (including the same time period)? Were inclusion and exclusion criteria for being in the study prespecified and applied uniformly to all participants?	Y	Y	Y	Y	Y	Y	Y	Y	Y	Y	Y	Y	Y	Y	Y	Y	Y
5. Was a sample size justification, power description, or variance and effect estimates provided?	N	N	N	N	N	N	N	N	N	N	N	N	N	N	N	N	N
6. For the analyses in this paper, were the exposure(s) of interest measured prior to the outcome(s) being measured?	Y	Y	Y	Y	Y	Y	Y	Y	Y	Y	Y	Y	Y	Y	Y	Y	Y

7. Was the timeframe sufficient so that one could reasonably expect to see an association between exposure and outcome if it existed?	N	Y	N	Y	N	Y	Y	Y	Y	Y	Y	N	Y	Y	Y	Y	Y
8. For exposures that can vary in amount or level, did the study examine different levels of the exposure as related to the outcome (e.g., categories of exposure, or exposure measured as continuous variable)?	Y	N	N	Y	N	N	N	N	N	N	N	N	N	N	N	N	N
9. Were the exposure measures (independent variables) clearly defined, valid, reliable, and implemented consistently across all study participants?	Y	Y	Y	Y	Y	Y	Y	Y	Y	Y	Y	Y	Y	Y	Y	Y	Y
10. Was the exposure(s) assessed more than once over time?	N	N	N	N	N	N	N	N	N	N	N	N	N	N	N	N	N
11. Were the outcome measures (dependent variables) clearly defined, valid, reliable, and implemented consistently across all study participants?	Y	Y	Y	Y	Y	Y	Y	Y	Y	Y	Y	Y	Y	Y	Y	Y	Y
12. Were the outcome assessors blinded to the exposure status of participants?	N	N**	N	N	N	N	N	N*	N*	N	N	N	N	N	N	N*	N
13. Was loss to follow-up after baseline 20% or less?	Y	Y	Y	Y	Y	Y	Y	Y	Y	Y	Y	Y	Y	Y	Y	Y	Y
14. Were key potential confounding variables measured and adjusted statistically for their impact on the relationship between exposure(s) and outcome(s)?	N	N	Y	Y	Y	Y	N	N	N	N	N	N	N	N	Y	N	Y
**Summary**	Fair	Fair	Fair	Good	Fair	Fair	Fair	Fair	Fair	Fair	Fair	Fair	Fair	Fair	Fair	Fair	Fair

Abbreviations: Y= YES, N= NO

Note: * = the outcome assessors were blinded to clinical assessments, ** = the outcome assessor was blinded to clinical assessments as well as patient treatment characteristics.

Quality Rating (Good, Fair, or Poor): Quality was rated as poor (0–4 out of 14 questions), fair (5–10 out of 14 questions), or good (11–14 out of 14 questions).
